# Methyl 4-{[(4-methyl­phen­yl)sulfon­yl]amino}­benzoate

**DOI:** 10.1107/S160053681200476X

**Published:** 2012-02-10

**Authors:** Muhammad Sohail, Muhammad Nadeem Asghar, M. Nawaz Tahir, Muhammad Shafique, Muhammad Ashfaq

**Affiliations:** a Forman Christian College (A Chartered University), Ferozepur Road, Lahore 54600, Pakistan; b Department of Physics, University of Sargodha, Sargodha, Pakistan; c Department of Chemistry, GC University, Lahore 54000, Pakistan; d Department of Chemistry, University of Gujrat, Gujrat, Pakistan

## Abstract

In the mol­ecule of the title compound, C_15_H_15_NO_4_S, the dihedral angle between the two rings is 88.05 (7)°. The methyl ester group is nearly coplanar with the adjacent ring [dihedral angle = 2.81 (10)°], whereas it is oriented at 86.90 (9)° with respect to the plane of the ring attached to the –SO_2_– group. Weak intra­molecular C—H⋯O hydrogen bonding completes *S*(5) and *S*(6) ring motifs. The mol­ecules form one-dimensional polymeric *C*(8) chains along the [010] direction due to N—H⋯O hydrogen bonding and these chains are linked by C—H⋯O hydrogen bonds, forming a three-dimensional network.

## Related literature

For related crystal structures, see: Mustafa *et al.* (2011 ▶); Nan & Xing (2006 ▶); Xing & Nan (2005 ▶). For graph-set notation, see: Bernstein *et al.* (1995 ▶).
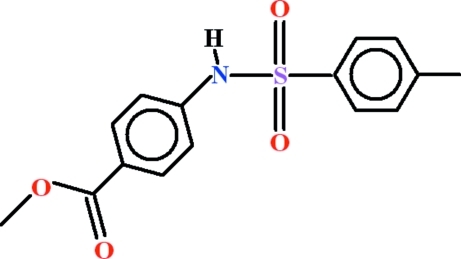


## Experimental

### 

#### Crystal data

C_15_H_15_NO_4_S
*M* *_r_* = 305.34
Monoclinic, 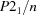

*a* = 7.9332 (2) Å
*b* = 8.2265 (2) Å
*c* = 22.7419 (5) Å
β = 92.769 (1)°
*V* = 1482.46 (6) Å^3^
*Z* = 4
Mo *K*α radiation
μ = 0.23 mm^−1^
*T* = 296 K
0.35 × 0.25 × 0.22 mm

#### Data collection

Bruker Kappa APEXII CCD diffractometer
Absorption correction: multi-scan (*SADABS*; Bruker, 2005 ▶) *T*_min_ = 0.915, *T*_max_ = 0.938
11165 measured reflections
2677 independent reflections
2234 reflections with *I* > 2σ(*I*)
*R*_int_ = 0.020

#### Refinement

*R*[*F* ^2^ > 2σ(*F* ^2^)] = 0.039
*wR*(*F* ^2^) = 0.113
*S* = 1.05
2677 reflections
192 parameters
H-atom parameters constrained
Δρ_max_ = 0.28 e Å^−3^
Δρ_min_ = −0.27 e Å^−3^

### 

Data collection: *APEX2* (Bruker, 2009 ▶); cell refinement: *SAINT* (Bruker, 2009 ▶); data reduction: *SAINT*; program(s) used to solve structure: *SHELXS97* (Sheldrick, 2008 ▶); program(s) used to refine structure: *SHELXL97* (Sheldrick, 2008 ▶); molecular graphics: *ORTEP-3 for Windows* (Farrugia, 1997 ▶) and *PLATON* (Spek, 2009 ▶); software used to prepare material for publication: *WinGX* (Farrugia, 1999 ▶) and *PLATON*.

## Supplementary Material

Crystal structure: contains datablock(s) global, I. DOI: 10.1107/S160053681200476X/bq2337sup1.cif

Structure factors: contains datablock(s) I. DOI: 10.1107/S160053681200476X/bq2337Isup2.hkl

Supplementary material file. DOI: 10.1107/S160053681200476X/bq2337Isup3.cml

Additional supplementary materials:  crystallographic information; 3D view; checkCIF report

## Figures and Tables

**Table 1:** Hydrogen-bond geometry (Å, °)

*D*—H⋯*A*	*D*—H	H⋯*A*	*D*⋯*A*	*D*—H⋯*A*
N1—H1⋯O3^i^	0.86	2.1800	2.878 (2)	138
C2—H2⋯O1	0.93	2.5200	2.898 (3)	105
C3—H3⋯O2^ii^	0.93	2.5500	3.431 (3)	159
C7—H7*C*⋯O1^iii^	0.96	2.5700	3.396 (3)	145
C9—H9⋯O1	0.93	2.3600	3.009 (3)	126
C10—H10⋯O2^iv^	0.93	2.5400	3.456 (2)	166
C15—H15*A*⋯O1^v^	0.96	2.5300	3.463 (3)	162

Symmetry codes: (i) 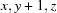; (ii) 
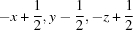
; (iii) 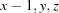; (iv) 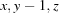; (v) 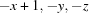.

**Fig. 1.**
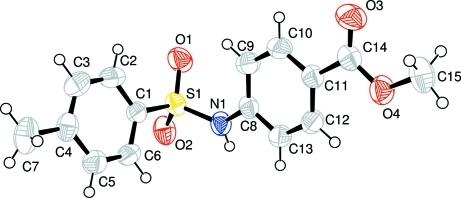
View of the title compound with the atom numbering scheme. The thermal ellipsoids are drawn at the 50% probability level.

**Fig. 2.**
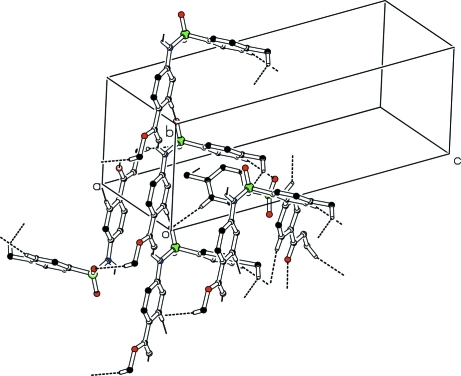
The partial packing (PLATON; Spek, 2009) which shows that molecules form polymeric chains extending along [010] direction. The H atoms not involved in H-bondings are omitted for clarity.

## supplementary crystallographic information

### Comment

The title compound (I), (Fig. 1) has been synthesized as a part of the series of new sulfonamide derivatives. The aim of our research work is to find the potential sulfonamide derivatives possessing anti-microbial *etc*.

The crystal structures of (II) *i.e*, 4-(((4-methylphenyl)sulfonyl)amino)benzoic acid (Mustafa *et al.* 2011; Nan & Xing, 2006) and (III) *i.e.**N*-(4-(ethoxycarbonyl)phenyl)-*p*-tolylsulfonamide (Xing & Nan, 2005) have been published which are related to (I).

In (I), the phenyl rings A (C1–C6) and B (C8—C13) are planar with r.m.s. deviation of 0.0043 Å and 0.0039 Å, respectively. The dihedral angle between A/B is 88.05 (7)°. The methyl ester moiety C (O3/C14/O4/C15) is also planar with r.m.s. deviation of 0.0015 Å. The dihedral angle between A/C and B/C is 86.90 (9)° and 2.81 (10)°, respectively. There exist intramolecular H-bonding of C—H···O type (Table 1, Fig. 1) forming an S(5) and S(6) ring motifs (Bernstein *et al.*, 1995). There exist also intermolecular H-bondings of N—H···O type (Table 1, Fig. 2) due to which the molecules form C(8) one-dimensional polymeric chains extending along the [010] direction. There exist *R*_2_^2^(9) ring motifs due to intermolecular H-bondings of C—H···O and N—H···O types (Table 1, Fig. 2). The other intermolecular H-bondings of C—H···O type interlink these polymeric chains to stabilize the molecules in the form of three-dimensional polymeric network.

### Experimental

2-(Diethylamino)ethyl 4-aminobenzoate hydrogen chloride (2.728 g, 10 mmol) was dissolved in distilled water (20 ml). The pH of the solution was maintained strictly at 8 to 9 using 1 *M* Na_2_CO_3_ solution. 4-Methyl sulfonyl chloride (1.906 g, 10 mmol) was then added to the solution while stirring at room temperature. On completion of the reaction the pH was adjusted to 1–2, using 1 N HCl while stirring. The precipitates obtained were filtered off, washed with distilled water, dried and subjected to re-crystallization using methanol to afford colorless prisms of (I). m.p. 343 K.

### Refinement

The H atoms were positioned geometrically (N—H = 0.86 Å, C—H = 0.93–0.96 Å) and refined as riding with *U*_iso_(H) = *xU*_eq_(C, N), where *x* = 1.5 for methyl groups and *x* = 1.2 for all H atoms.

### Crystal data

**Table:**

C_15_H_15_NO_4_S	*F*(000) = 640
*M**_r_* = 305.34	*D*_x_ = 1.368 Mg m^−^^3^
Monoclinic, *P*2_1_/*n*	Mo *K*α radiation, λ = 0.71073 Å
Hall symbol: -P 2yn	Cell parameters from 2488 reflections
*a* = 7.9332 (2) Å	θ = 2.6–25.3°
*b* = 8.2265 (2) Å	µ = 0.23 mm^−^^1^
*c* = 22.7419 (5) Å	*T* = 296 K
β = 92.769 (1)°	Prism, colourless
*V* = 1482.46 (6) Å^3^	0.35 × 0.25 × 0.22 mm
*Z* = 4	

### Data collection

**Table:**

Bruker Kappa APEXII CCD diffractometer	2677 independent reflections
Radiation source: fine-focus sealed tube	2234 reflections with *I* > 2σ(*I*)
Graphite monochromator	*R*_int_ = 0.020
Detector resolution: 8.00 pixels mm^-1^	θ_max_ = 25.3°, θ_min_ = 2.6°
ω scans	*h* = −9→9
Absorption correction: multi-scan (*SADABS*; Bruker, 2005)	*k* = −9→9
*T*_min_ = 0.915, *T*_max_ = 0.938	*l* = −27→27
11165 measured reflections	

### Refinement

**Table:**

Refinement on *F*^2^	Primary atom site location: structure-invariant direct methods
Least-squares matrix: full	Secondary atom site location: difference Fourier map
*R*[*F*^2^ > 2σ(*F*^2^)] = 0.039	Hydrogen site location: inferred from neighbouring sites
*wR*(*F*^2^) = 0.113	H-atom parameters constrained
*S* = 1.05	*w* = 1/[σ^2^(*F*_o_^2^) + (0.0548*P*)^2^ + 0.5778*P*] where *P* = (*F*_o_^2^ + 2*F*_c_^2^)/3
2677 reflections	(Δ/σ)_max_ < 0.001
192 parameters	Δρ_max_ = 0.28 e Å^−^^3^
0 restraints	Δρ_min_ = −0.27 e Å^−^^3^

### Special details

**Table:**

Geometry. Bond distances, angles *etc*. have been calculated using the rounded fractional coordinates. All su's are estimated from the variances of the (full) variance-covariance matrix. The cell e.s.d.'s are taken into account in the estimation of distances, angles and torsion angles
Refinement. Refinement of *F*^2^ against ALL reflections. The weighted *R*-factor *wR* and goodness of fit *S* are based on *F*^2^, conventional *R*-factors *R* are based on *F*, with *F* set to zero for negative *F*^2^. The threshold expression of *F*^2^ > σ(*F*^2^) is used only for calculating *R*-factors(gt) *etc*. and is not relevant to the choice of reflections for refinement. *R*-factors based on *F*^2^ are statistically about twice as large as those based on *F*, and *R*-factors based on ALL data will be even larger.

### Fractional atomic coordinates and isotropic or equivalent isotropic displacement parameters (Å^2^)

**Table:**

	*x*	*y*	*z*	*U*_iso_*/*U*_eq_	
S1	0.44553 (6)	0.55050 (6)	0.13445 (2)	0.0511 (2)	
O1	0.55975 (19)	0.43449 (19)	0.16030 (7)	0.0678 (5)	
O2	0.4968 (2)	0.71630 (18)	0.12957 (7)	0.0652 (5)	
O3	0.2286 (2)	−0.24936 (18)	0.00003 (8)	0.0721 (6)	
O4	0.1308 (2)	−0.11198 (18)	−0.07868 (7)	0.0622 (5)	
N1	0.3940 (2)	0.4952 (2)	0.06731 (7)	0.0521 (5)	
C1	0.2595 (2)	0.5454 (2)	0.17369 (8)	0.0449 (6)	
C2	0.2551 (3)	0.4587 (3)	0.22508 (10)	0.0659 (8)	
C3	0.1124 (3)	0.4643 (3)	0.25712 (10)	0.0711 (9)	
C4	−0.0272 (3)	0.5533 (3)	0.23879 (9)	0.0552 (7)	
C5	−0.0206 (3)	0.6378 (3)	0.18633 (9)	0.0586 (7)	
C6	0.1211 (3)	0.6352 (3)	0.15395 (9)	0.0538 (6)	
C7	−0.1815 (3)	0.5595 (4)	0.27480 (11)	0.0781 (10)	
C8	0.3434 (2)	0.3396 (2)	0.04751 (8)	0.0451 (6)	
C9	0.3805 (3)	0.1972 (3)	0.07832 (10)	0.0588 (7)	
C10	0.3323 (3)	0.0495 (2)	0.05412 (9)	0.0574 (7)	
C11	0.2481 (2)	0.0400 (2)	−0.00030 (8)	0.0466 (6)	
C12	0.2112 (3)	0.1827 (3)	−0.03051 (9)	0.0536 (6)	
C13	0.2571 (3)	0.3307 (2)	−0.00672 (9)	0.0525 (6)	
C14	0.2038 (3)	−0.1214 (3)	−0.02501 (9)	0.0520 (7)	
C15	0.0800 (3)	−0.2637 (3)	−0.10630 (12)	0.0721 (9)	
H1	0.39816	0.56949	0.04082	0.0625*	
H2	0.34755	0.39672	0.23815	0.0791*	
H3	0.11033	0.40628	0.29218	0.0853*	
H5	−0.11403	0.69750	0.17272	0.0703*	
H6	0.12372	0.69359	0.11898	0.0645*	
H7A	−0.22071	0.66961	0.27694	0.1174*	
H7B	−0.15350	0.52002	0.31379	0.1174*	
H7C	−0.26853	0.49272	0.25664	0.1174*	
H9	0.43730	0.20136	0.11503	0.0705*	
H10	0.35718	−0.04558	0.07491	0.0688*	
H12	0.15469	0.17832	−0.06728	0.0642*	
H13	0.22991	0.42566	−0.02725	0.0630*	
H15A	0.17843	−0.32685	−0.11387	0.1082*	
H15B	0.01810	−0.24183	−0.14275	0.1082*	
H15C	0.00991	−0.32322	−0.08062	0.1082*	

### Atomic displacement parameters (Å^2^)

**Table:**

	*U*^11^	*U*^22^	*U*^33^	*U*^12^	*U*^13^	*U*^23^
S1	0.0506 (3)	0.0488 (3)	0.0540 (3)	−0.0005 (2)	0.0037 (2)	−0.0092 (2)
O1	0.0567 (8)	0.0731 (10)	0.0721 (10)	0.0153 (7)	−0.0114 (7)	−0.0152 (8)
O2	0.0702 (9)	0.0558 (9)	0.0707 (10)	−0.0156 (7)	0.0161 (8)	−0.0158 (7)
O3	0.0901 (12)	0.0417 (9)	0.0834 (12)	−0.0012 (8)	−0.0057 (9)	0.0020 (8)
O4	0.0744 (10)	0.0548 (9)	0.0572 (9)	−0.0059 (7)	0.0018 (7)	−0.0106 (7)
N1	0.0674 (10)	0.0418 (9)	0.0478 (9)	−0.0040 (8)	0.0107 (8)	−0.0028 (7)
C1	0.0516 (10)	0.0397 (10)	0.0432 (10)	−0.0008 (8)	0.0008 (8)	−0.0022 (8)
C2	0.0750 (14)	0.0629 (14)	0.0600 (13)	0.0175 (11)	0.0052 (11)	0.0180 (11)
C3	0.0922 (17)	0.0678 (15)	0.0543 (13)	0.0031 (13)	0.0145 (12)	0.0197 (11)
C4	0.0620 (12)	0.0560 (12)	0.0480 (11)	−0.0152 (10)	0.0081 (9)	−0.0109 (9)
C5	0.0540 (11)	0.0682 (14)	0.0534 (12)	0.0063 (10)	0.0005 (9)	0.0017 (10)
C6	0.0584 (11)	0.0590 (12)	0.0439 (10)	0.0046 (10)	0.0018 (9)	0.0095 (9)
C7	0.0740 (15)	0.097 (2)	0.0652 (15)	−0.0211 (14)	0.0218 (12)	−0.0148 (14)
C8	0.0479 (10)	0.0424 (10)	0.0460 (10)	0.0000 (8)	0.0127 (8)	−0.0030 (8)
C9	0.0733 (14)	0.0487 (12)	0.0532 (12)	0.0034 (10)	−0.0090 (10)	−0.0004 (9)
C10	0.0723 (13)	0.0408 (11)	0.0583 (12)	0.0038 (9)	−0.0037 (10)	0.0050 (9)
C11	0.0484 (10)	0.0434 (10)	0.0486 (10)	0.0000 (8)	0.0082 (8)	−0.0015 (8)
C12	0.0671 (12)	0.0503 (11)	0.0431 (10)	−0.0013 (10)	0.0005 (9)	0.0009 (9)
C13	0.0678 (12)	0.0420 (10)	0.0478 (11)	0.0007 (9)	0.0039 (9)	0.0050 (9)
C14	0.0512 (11)	0.0482 (12)	0.0573 (12)	−0.0006 (9)	0.0095 (9)	−0.0038 (10)
C15	0.0735 (15)	0.0665 (15)	0.0773 (16)	−0.0151 (12)	0.0126 (12)	−0.0254 (13)

### Geometric parameters (Å, º)

**Table:**

S1—O1	1.4234 (16)	C10—C11	1.380 (3)
S1—O2	1.4292 (16)	C11—C14	1.478 (3)
S1—N1	1.6264 (17)	C11—C12	1.384 (3)
S1—C1	1.7617 (17)	C12—C13	1.374 (3)
O3—C14	1.208 (3)	C2—H2	0.9300
O4—C14	1.328 (3)	C3—H3	0.9300
O4—C15	1.446 (3)	C5—H5	0.9300
N1—C8	1.409 (2)	C6—H6	0.9300
N1—H1	0.8600	C7—H7A	0.9600
C1—C6	1.380 (3)	C7—H7B	0.9600
C1—C2	1.371 (3)	C7—H7C	0.9600
C2—C3	1.376 (3)	C9—H9	0.9300
C3—C4	1.375 (3)	C10—H10	0.9300
C4—C7	1.506 (3)	C12—H12	0.9300
C4—C5	1.384 (3)	C13—H13	0.9300
C5—C6	1.374 (3)	C15—H15A	0.9600
C8—C13	1.383 (3)	C15—H15B	0.9600
C8—C9	1.389 (3)	C15—H15C	0.9600
C9—C10	1.380 (3)		
			
O1—S1—O2	119.61 (9)	O3—C14—O4	122.5 (2)
O1—S1—N1	109.05 (9)	O3—C14—C11	124.98 (19)
O1—S1—C1	107.88 (9)	C1—C2—H2	120.00
O2—S1—N1	104.69 (9)	C3—C2—H2	120.00
O2—S1—C1	108.10 (9)	C2—C3—H3	119.00
N1—S1—C1	106.87 (8)	C4—C3—H3	119.00
C14—O4—C15	116.68 (18)	C4—C5—H5	119.00
S1—N1—C8	127.52 (13)	C6—C5—H5	119.00
S1—N1—H1	116.00	C1—C6—H6	120.00
C8—N1—H1	116.00	C5—C6—H6	120.00
S1—C1—C2	120.04 (15)	C4—C7—H7A	109.00
C2—C1—C6	120.20 (18)	C4—C7—H7B	109.00
S1—C1—C6	119.68 (15)	C4—C7—H7C	109.00
C1—C2—C3	119.3 (2)	H7A—C7—H7B	109.00
C2—C3—C4	121.9 (2)	H7A—C7—H7C	110.00
C5—C4—C7	121.2 (2)	H7B—C7—H7C	109.00
C3—C4—C5	117.7 (2)	C8—C9—H9	120.00
C3—C4—C7	121.1 (2)	C10—C9—H9	120.00
C4—C5—C6	121.4 (2)	C9—C10—H10	119.00
C1—C6—C5	119.5 (2)	C11—C10—H10	119.00
N1—C8—C13	117.01 (15)	C11—C12—H12	120.00
C9—C8—C13	119.26 (17)	C13—C12—H12	120.00
N1—C8—C9	123.70 (17)	C8—C13—H13	120.00
C8—C9—C10	119.6 (2)	C12—C13—H13	120.00
C9—C10—C11	121.35 (18)	O4—C15—H15A	109.00
C10—C11—C12	118.60 (17)	O4—C15—H15B	109.00
C12—C11—C14	122.18 (18)	O4—C15—H15C	109.00
C10—C11—C14	119.21 (16)	H15A—C15—H15B	109.00
C11—C12—C13	120.68 (19)	H15A—C15—H15C	109.00
C8—C13—C12	120.54 (17)	H15B—C15—H15C	110.00
O4—C14—C11	112.48 (19)		
			
O1—S1—N1—C8	47.14 (18)	C2—C3—C4—C7	−179.3 (2)
O2—S1—N1—C8	176.25 (15)	C3—C4—C5—C6	−0.9 (4)
C1—S1—N1—C8	−69.23 (17)	C7—C4—C5—C6	178.7 (2)
O1—S1—C1—C2	9.46 (19)	C4—C5—C6—C1	0.6 (4)
O1—S1—C1—C6	−173.61 (16)	N1—C8—C9—C10	−177.01 (19)
O2—S1—C1—C2	−121.20 (17)	C13—C8—C9—C10	0.7 (3)
O2—S1—C1—C6	55.73 (18)	N1—C8—C13—C12	176.58 (19)
N1—S1—C1—C2	126.60 (17)	C9—C8—C13—C12	−1.2 (3)
N1—S1—C1—C6	−56.47 (18)	C8—C9—C10—C11	0.2 (3)
C15—O4—C14—O3	0.5 (3)	C9—C10—C11—C12	−0.4 (3)
C15—O4—C14—C11	−178.99 (17)	C9—C10—C11—C14	178.2 (2)
S1—N1—C8—C9	−21.7 (3)	C10—C11—C12—C13	−0.2 (3)
S1—N1—C8—C13	160.56 (16)	C14—C11—C12—C13	−178.7 (2)
S1—C1—C2—C3	175.92 (17)	C10—C11—C14—O3	3.6 (3)
C6—C1—C2—C3	−1.0 (3)	C10—C11—C14—O4	−176.90 (19)
S1—C1—C6—C5	−176.55 (17)	C12—C11—C14—O3	−177.8 (2)
C2—C1—C6—C5	0.4 (3)	C12—C11—C14—O4	1.7 (3)
C1—C2—C3—C4	0.7 (4)	C11—C12—C13—C8	1.0 (3)
C2—C3—C4—C5	0.3 (4)		

### Hydrogen-bond geometry (Å, º)

**Table:**

*D*—H···*A*	*D*—H	H···*A*	*D*···*A*	*D*—H···*A*
N1—H1···O3^i^	0.86	2.1800	2.878 (2)	138
C2—H2···O1	0.93	2.5200	2.898 (3)	105
C3—H3···O2^ii^	0.93	2.5500	3.431 (3)	159
C7—H7*C*···O1^iii^	0.96	2.5700	3.396 (3)	145
C9—H9···O1	0.93	2.3600	3.009 (3)	126
C10—H10···O2^iv^	0.93	2.5400	3.456 (2)	166
C15—H15*A*···O1^v^	0.96	2.5300	3.463 (3)	162

Symmetry codes: (i) *x*, *y*+1, *z*; (ii) −*x*+1/2, *y*−1/2, −*z*+1/2; (iii) *x*−1, *y*, *z*; (iv) *x*, *y*−1, *z*; (v) −*x*+1, −*y*, −*z*.
